# The impact of X chromosome inactivation on human health

**DOI:** 10.3389/fgene.2026.1845863

**Published:** 2026-05-29

**Authors:** Junyi Gu, Qian Chen

**Affiliations:** 1 Department of Reproductive Medicine, The First Affiliated Hospital of Xi’an Jiaotong University, Xi’an, Shaanxi, China; 2 School of Medicine, Xi’an Jiaotong University, Xi’an, Shaanxi, China

**Keywords:** health impact, sex differences, therapeutic strategies, X chromosome inactivation, XCI escape, XCI skewing

## Abstract

X chromosome inactivation (XCI) is a crucial genetic regulatory mechanism in female cells, wherein one of the two X chromosomes is randomly silenced to balance gene expression between sexes. Despite the overall silencing, approximately 15%–30% of human genes escape XCI, leading to their biallelic expression in females. Many studies have highlighted the phenomenon of XCI skewing, where the inactivation is preferentially biased towards one X chromosome. XCI escape and XCI skewing are significantly associated with various health issues, including autoimmune disorders, neurodevelopmental disorders (NDDS), cardiovascular diseases (CVD) and cancer, etc. This review aims to provide a comprehensive overview of the mechanisms underlying XCI, its roles across different genders and disease states, and to explore the intricate relationship between XCI and human health. By emphasizing the significance of understanding XCI, this article seeks to shed light on potential therapeutic targets and avenues for further research in the field.

## 1 Introduction

The sex is determined by sex chromosomes in a range of animal lineages, including nematodes, insects, and mammals. A fundamental consequence of sex chromosome evolution is the emergence of disparate gene dosages between the sexes. In humans, female somatic cells contain two X chromosomes, while males possess one X and one Y chromosome. Consequently, this chromosomal difference creates a twofold disparity in the dosage of X-linked genes between the sexes. To resolve this discrepancy, female somatic cells employ X chromosome inactivation (XCI), a process that transcriptionally silences one X chromosome to achieve dosage equivalency for X-linked genes between XY males and XX females. Originally hypothesized by Lyon (1961), XCI is established as early as the eight-cell stage during female embryonic development (Disteche and Berletch, 2015), and the fixed inactivation is then inherited through subsequent divisions in somatic cells. Ultimately, XCI balances gene dosage by ensuring that only one X chromosome is transcriptionally active per diploid cell (Fang et al., 2019).

Despite the overall silencing, approximately 15%**–**30% of human genes escape XCI, resulting in biallelic expression in females (Peeters et al., 2023; Topa et al., 2024). These escape genes are not uniformly distributed and can vary by tissue type, developmental stage, and individual, contributing to mosaicism in X-linked gene expression (Naik et al., 2022). The differential expression of escape genes accounts for sex differences in gene dosage and can influence susceptibility to various diseases, particularly those with a known female bias such as systemic lupus erythematosus (SLE) (Dossin et al., 2020). Biallelic expression of *TLR7* in female immune cells enhances immune responsiveness and may predispose to autoimmunity (Rodermund et al., 2021; Tjalsma et al., 2021). Beyond autoimmunity, XCI escape also influences other disease phenotypes and sex differences. In cancer, certain tumor suppressor genes (TSGs) located on the X chromosome escape inactivation and may contribute to the lower incidence of some cancers in females compared to males (Wang et al., 2020). In female alveolar type 2 cells, defective maintenance of XCI leads to the escape of X-linked genes such as *ACE2*, which in turn drives sex-biased gene expression and may underlie sex differences in lung diseases (Sierra et al., 2023). Even in rare X-linked disorders, such as choroideremia, XCI escape and the resulting biallelic expression can modify disease manifestation in female carriers, challenging the traditional view of X-linked recessive inheritance (Di Giosaffatte et al., 2022).

XCI skewing describes the phenomenon when more than 75% of cells in an individual chose the X chromosome from one parent as the inactive X chromosome (Xi) (Dardik et al., 2021). Under normal conditions, which X chromosome is silenced is completely random, with each having a 50% probability. However, when a specific allele from one parent confers greater cellular robustness, this randomness is disrupted, leading to XCI skewing (Migeon and Haisley-Royster, 1998). It is estimated that 1.5%–23% of females have XCI skewing (Cantone and Fisher, 2017). The clinical relevance of XCI skewing is profound. It can unmask deleterious variants on the Xi, leading to symptomatic manifestations in female carriers of X-linked diseases such as Duchenne/Becker muscular dystrophy, hemophilia, chronic granulomatous disease, and intellectual disability (Huang et al., 2023; Korotkova et al., 2025; Shoukat et al., 2020; Zhang et al., 2024). Furthermore, an uneven pattern of XCI has been correlated with a higher incidence of autoimmune diseases in females, possibly due to the varied expression of immune-related escape genes located on the X chromosome (Scofield et al., 2025). In the realm of oncology, irregular XCI patterns and evasion from inactivation have been associated with tumor development and differences in cancer susceptibility based on sex (Sadagopan et al., 2022). In summary, XCI is a fundamental process that has far-reaching implications for gene expression, health, and disease in females. The random nature of XCI, the phenomenon of gene escape, and the potential consequences of XCI skewing patterns are critical areas of research that continue to unveil the complexities of sex differences in biology and medicine.

This review aims to comprehensively summarize the current concepts, characteristics, causes, and molecular mechanisms of XCI escape and skewing. We will also explore their impact on human health, emphasizing its implications in disease susceptibility and clinical outcomes. By integrating recent advances from molecular biology, genetics, and clinical research, this overview seeks to provide a coherent framework to guide future investigations and therapeutic strategies targeting XCI-related phenomena.

## 2 Basic mechanisms of XCI

### 2.1 Initiation, establishment and maintenance of XCI

**FIGURE 1:**
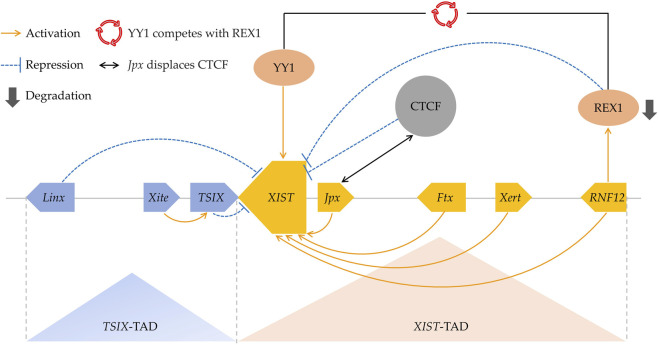
Regulation of X-chromosome inactivation (XCI). The *TSIX*-TAD and *XIST*-TAD refer to the topologically associating region of *TSIX* and *XIST* on the X chromosome, respectively.

The initiation of XCI is a critical process that start at the X-inactivation center (Xic), located at Xq13.1 - Xq13.2. The key element was identified as a gene that encodes a 17 kb lncRNA known as the X inactive-specific transcript (*XIST*) (Disteche and Berletch, 2015). *XIST* is upregulated in early embryonic development, marking the transition towards XCI. This upregulation is subject to tight regulation by both positive and negative feedback mechanisms. The positive regulators of *XIST* encoded by the X chromosome have been identified, comprising the RING finger protein 12 gene (*RNF12*), non-coding RNAs such as Ftx, Jpx, Linx, the XIST-enhancing regulatory transcript (*Xert*), and etc (Barakat et al., 2011; Chureau et al., 2011; Ravid Lustig et al., 2023). The expression of these factors is upregulated during early differentiation, which partially explains their role in initiating XCI. In contrast, the *XIST* antisense non-coding transcription unit *TSIX* transcribed in the opposite direction is the most prominent negative regulator of *XIST*. Mutations in *TSIX* leads to abnormal *XIST* upregulation. The *Xite* enhancer drives *TSIX* transcription, which in turn suppresses the transcription of *XIST*. The CCCTC-binding factor (CTCF) represses the *XIST* transcription, while *Jpx* activates it by displacing CTCF. The degradation of the pluripotency factor REX1 relieves the repression of *XIST*, thereby leading to its upregulation. In addition,the transcription factor Yin Yang 1 (YY1) competes with REX1 for binding to the 5′region of *XIST*, thereby activating the *XIST* promoter (Figure 1).

The next wave of XCI is the establishment of silencing on Xi, which entails multiple steps, ensuing with the spreading and coating of the *XIST* on Xi. The repeat A region of *XIST*. RNA plays a pivotal role in this process by interacting with the SPlit ENds (SPEN) protein, receptor corepressor (NCoR), silencing mediator of retinoic acid and thyroid hormone receptor (SMRT), and nucleosome remodeling deacetylase (NuRD) to mediate histone deacetylation (Dossin et al., 2020; Jachowicz et al., 2022; McHugh et al., 2015; Żylicz et al., 2019), while also establishing N6-methyladenosine (m^6^A) methylation through recruitment of proteins such as RNA-binding motif proteins 15 (RBM15) (Coker et al., 2020). These cascading mechanisms collectively achieve transcriptional silencing of nearly all genes on the Xi transcriptional silencing. Repeat B and C maintain Xi in its inactive state by recruiting polycomb repressive complexes 1 (PRC1) and 2 (PRC2) (Plath et al., 2004; Schoeftner et al., 2006). These proteins remodel the epigenetic landscape of Xi by introducing repressive marks, including H3K27me3, H3K9me2, H4K20me1, and H2Aub1, modifications that are essential for stabilizing the silenced state (Costanzi and Pehrson, 1998; Heard et al., 2001; Plath et al., 2003; Rougeulle et al., 2004). The absence of these marks can lead to the loss of XCI and aberrant expression of X-linked genes, which has been implicated in various diseases, including autoimmune disorders and cancers (Chlamydas et al., 2022; Pyfrom et al., 2021). Four proteins, polypyrimidine tract-binding protein 1(PTBP1), Matrin 3 (MATR3), TAR DNA-binding protein 43 (TDP-43), and CUGBP Elav-like family member 1 (CELF1), interact with Repeat E, forming a CIZ1-independent complex that stabilizes *XIST* RNA. In summary, the plethora of epigenetic modifications accumulating on Xi define its higher-order organization, which is essential for stabilizing this dimensional structure of Xi. In addition, late-stage epigenetic modifications during the maintenance phase of XCI also include the specific enrichment of the histone variant macroH2A on the Xi (Costanzi et al., 2000), CpG island DNA methylation mediated by DNA (cytosine-5)-methyltransferase 3B (DNMT3B) (Gendrel et al., 2012). However, the precise roles of these late-stage epigenetic modifications in XCI maintenance and their dependence on *XIST* RNA remain unclear.

### 2.2 Mechanisms of XCI escape and XCI skewing

#### 2.2.1 Molecular characteristics and regulatory mechanisms of escape genes

Escape genes are broadly categorized as either constitutive, escaping XCI ubiquitously across tissues and individuals, or facultative, demonstrating escape patterns that vary by tissue or individual (Balaton et al., 2021; Zito et al., 2023). Escape genes exhibit a non-random distribution pattern on the X chromosome, primarily enriching in the distal region of the short arm, accounting for about 21% of the total number, and only 3% are located in the long arm. These regions display unique chromatin accessibility characteristics, with the chromatin openness of the distal regions significantly increasing during aging (Fang et al., 2025; Hoelzl et al., 2025). Most human X-chromosome escape genes have homologous sequences on the Y chromosome and are clustered in the pseudoautosomal region (PAR) at the distal end of Xp. Examples include *XG* (Xg blood group gene), *MIC2* (encoding the cell surface antigen CD99), and *ANT3* (mediating ADP/ATP exchange). Additionally, a small number of XCI escape genes that lack Y-chromosome homologs have also been identified; these genes are distributed near the PAR, such as *STS* and *KALP* (Berletch et al., 2010). Cross-species comparisons reveal that while the majority of genes subject to XCI are conserved, certain genes exhibit species-specific escape patterns, highlighting evolutionary divergence in XCI regulation (Raposo et al., 2025). Functionally, escape genes are enriched in pathways related to immunity, neural development, and other critical biological processes, underscoring their importance in sex differences and disease susceptibility (Aguado et al., 2022; Balaton et al., 2021).

**FIGURE 2:**
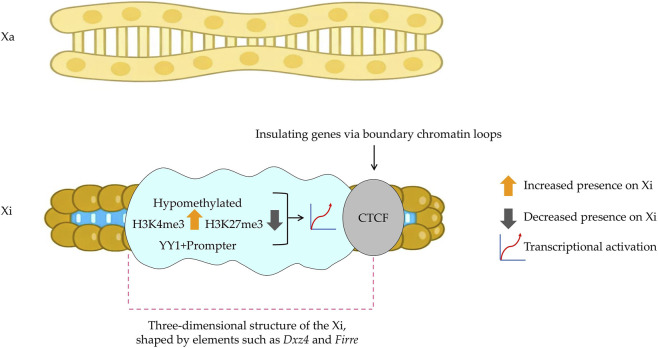
The molecular mechanisms underlying XCI escape. Xa refers to the active X chromosome. Xi refers to the inactive X chromosome.

The transcriptional regulation of escape genes is intricately controlled by epigenetic and chromatin features that distinguish them from fully inactivated genes. The promoter regions of escape genes are characteristically hypomethylated, thereby allowing for their continued transcription from the Xi (de Oliveira et al., 2025). For example, targeted demethylation of the *CDKL5* promoter using dCas9-TET1 fusion proteins has been shown to reactivate the inactive allele (Kawashima et al., 2021). Escape genes generally show reduced enrichment of repressive marks such as H_3_K_27_me_3_ and increased presence of active marks like H_3_K_4_me_3_ on the Xi (Chureau et al., 2011). These active marks recruit chromatin remodeling complexes like SWI/SNF, which promote an open chromatin conformation conducive to transcriptional initiation and elongation. On the Xi, escape genes are organized into discrete topologically associating domains (TADs) defined by CTCF and cohesin binding sites. Together, these architectural factors promote chromatin loop formation, physically segregating escape gene domains from adjacent silenced regions. Compared to the Xa, the Xi exhibits a more compact TAD structure overall, reflecting the global transcriptional repression characteristic of XCI. However, the TADs encompassing escape genes maintain a relatively open conformation, which permits access to transcription factors and RNA polymerase II, thereby enabling transcriptional activity despite the surrounding silencing environment (Bansal et al., 2019). The *Dxz4* and *Firre* loci are key architectural elements that contribute to the unique three-dimensional organization of the inactive X chromosome and the regulation of escape genes (Dossin and Heard, 2022). *Dxz4* acts as a boundary element that partitions the Xi into two large domains, effectively segregating escape gene-rich regions from silenced domains. *Firre* encodes a lncRNA whose gene locus also contains tandem repeats and CTCF binding sites. *Firre* RNA mediates chromatin interactions that cluster multiple escape genes within the same three-dimensional nuclear space, promoting their coordinated transcriptional activity. Functional studies have demonstrated that deletion of *Dxz4* or *Firre* disrupts the normal chromatin organization of the Xi, leading to altered escape gene expression and increased silencing. Specifically, loss of *Dxz4* abolishes the megadomain boundary and reduces insulation, whereas deletion of *Firre* diminishes the clustering of escape genes and decreases their transcriptional output. Furthermore, transcription factors enriched at escape gene promoters, such as YY1, contribute to escape by facilitating transcriptional activation despite the repressive environment (Brown et al., 1992; Peeters et al., 2023) (Figure 2).

In summary, XCI escape arises from an interdependent network of epigenetic and structural features, where DNA hypomethylation and active histone marks create open chromatin, while CTCF-mediated 3D organization insulates escape genes from global Xi silencing. This synergy ensures that escape genes are selectively expressed despite the global silencing of the Xi, highlighting the multilayered complexity of XCI escape regulation.

#### 2.2.2 Characteristics and mechanisms of XCI skewing

**FIGURE 3:**
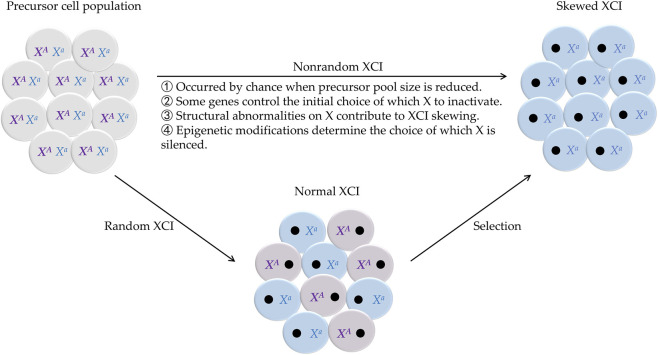
XCI skewing in females (adapted from Minks et al., 2008, with modifications). Xm and Xp denote the maternally and paternally derived X chromosomes, respectively, in female embryos. The deeply stained round within the cell represents the heterochromatic inactive X chromosome. The staining of the cells reflects the differential parental origin of their remaining active X chromosome.

XCI skewing can be categorized into primary skewing and secondary skewing. Primary skewing refers to the unequal probability of inactivating either of the two X chromosomes during the initial random establishment of XCI, driven by genetic or epigenetic variations. Its core mechanism lies in sequence polymorphisms within the XIC, such as single nucleotide polymorphisms (SNPs) or copy number variations (CNVs), which can directly influence the transcriptional regulation of the *XIST* gene. This bias is typically established during early embryonic development and is stably propagated through mitosis in subsequent cell divisions. Although primary skewing is commonly observed among individuals, its direct impact on tissue function is relatively limited. Only when combined with subsequent secondary selection mechanisms does it manifest significant phenotypic effects, particularly in the context of specific pathologies or aging. Secondary skewing arises after the initial random inactivation event and is driven by selective pressures that favor the expansion of cell clones harboring a particular active X chromosome. This phenomenon results in a deviation from the original random XCI pattern due to differential survival, proliferation, or functional advantages conferred by alleles on the active X chromosome (Figure 3).

Age-related XCI skewing arises from four interconnected mechanisms, including DNA methylation loss, histone dysregulation, somatic mutations, and mitochondrial dysfunction. On the Xi, promoter CpG methylation is essential for stable gene silencing. However, aging reduces the expression and activity of DNA methyltransferases such as DNMT1 and DNMT3A, leading to CpG hypomethylation on the Xi and subsequent destabilization of XCI patterns. This epigenetic drift accumulates over time and underlies the increased skewing observed in hematopoietic cells of elderly females (Li et al., 2020). Compounding this DNA methylation loss, the repressive chromatin environment maintained by histone modifications also deteriorates with age. The maintenance of XCI relies on multiple repressive marks, notably H3K27me3 catalyzed by the PRC2 complex, but aging cells exhibit decreased expression of EZH2, the catalytic subunit of PRC2, leading to reduced H3K27me3 deposition on the Xi (Shin et al., 2022). Such reduced H3K27me3 can weaken the heterochromatic barrier, rendering the Xi more susceptible to epigenetic instability and secondary skewing of XCI patterns (Chen et al., 2025). Beyond these epigenetic erosions, aging is characterized by the gradual accumulation of somatic mutations in tissue stem cells. When such mutations occur on the Xa and affect key genes involved in cell cycle regulation, tumor suppression, or apoptosis, the mutant clone can expand preferentially, leading to secondary skewing of XCI due to clonal hematopoiesis (Ayachi et al., 2020). This mechanism is particularly evident in the hematopoietic system. Finally, age-related mitochondrial dysfunction adds another layer of selective pressure. Several nuclear-encoded mitochondrial genes reside on the X chromosome, including *Timm17a* and *Ndufa1*, which are integral to mitochondrial protein import and electron transport chain function, respectively. The inactivation status of these genes on the Xi can influence cellular energy metabolism. If the Xa harbors alleles that confer superior mitochondrial function, cells expressing this Xa may gain a selective survival advantage during aging, promoting clonal expansion and contributing to secondary skewing of XCI (Errigo et al., 2023).

The biological basis of tissue-specific skewing involves stem cell dynamics and clonal selection pressure, among other factors. Stem cells from different tissues possess unique renewal rates and differentiation patterns, which directly influence the accumulation rate and extent of XCI skewing. Hematopoietic stem cells (HSCs), for instance, exhibit a high self-renewal capacity and continuous proliferation to maintain blood cell homeostasis, which predisposes the hematopoietic system to pronounced secondary XCI skewing over time. This is supported by evidence showing that aged HSCs display impaired maintenance of XCI, linked to alterations in nuclear architecture such as LaminA/C reduction, leading to increased chromatin accessibility and hypomethylation on the Xi (Grigoryan et al., 2021). The high turnover rate of HSCs facilitates clonal expansion of cells harboring advantageous active X alleles, thereby accelerating the accumulation of skewed XCI patterns. Conversely, neural stem cells (NSCs) in the brain have a much lower renewal rate, yet aging-related DNA damage accumulation can still induce localized XCI skewing, particularly in regions like the hippocampus and cortex where neurogenesis persists into adulthood (Xiong et al., 2020). Although NSCs divide less frequently, the cumulative effects of DNA damage and epigenetic drift may lead to selective pressures favoring certain X alleles, contributing to subtle but regionally specific XCI biases. The tissue microenvironment exerts a critical influence on the clonal selection pressures that shape XCI skewing, particularly during aging and pathological conditions. Changes in the microenvironment, including chronic inflammation, oxidative stress, and nutrient availability, can differentially affect cells depending on which X chromosome is active, thereby driving selective expansion of clones with advantageous active X alleles. For example, in chronic inflammatory tissues, immune cells expressing an active X chromosome harboring beneficial alleles of immune-related genes such as *TLR7* or *CXCR3* may gain a survival or functional advantage, leading to preferential clonal expansion and resultant XCI skewing (Roberts et al., 2024). Similarly, in the tumor microenvironment, the inactivation status of X-linked tumor suppressor genes such as *FOXP3* and *MECP2* can influence cell proliferation and survival, thereby driving clonal selection and tissue-specific XCI skewing (Bhangu et al., 2025).

It is worth noting that the presence of escape genes may indirectly drive XCI skewing by influencing cell proliferation or survival capacity. For example, if an escape gene such as *SHOX* in the PAR1 region exerts pro-proliferative effects in specific tissues, cell clones carrying the active allele may expand, leading to skewing. Furthermore, the expression levels of escape genes can be altered by XCI skewing. When a particular X chromosome predominates in a cell population, the expression of its escape genes increases, thereby affecting tissue function. Single-cell studies have revealed high heterogeneity in escape gene expression, a heterogeneity that may be amplified during aging, and together with XCI skewing, shape tissue-specific phenotypes. For instance, in female carriers of X-linked retinoschisis (XLRS), lyonization-induced differences in escape gene expression can cause subtle retinal structural alterations, such as thickening of the outer plexiform layer and reduction of the foveal avascular zone area (Tao et al., 2023). Thus, the bidirectional relationship between escape gene expression and XCI skewing constitutes a regulatory feedback loop that modulates gene dosage, cellular fitness, and tissue homeostasis.

## 3 Human disease driven by XCI abnormality

### 3.1 Autoimmune diseases

The X chromosome is rich in immune-related genes, and its escape phenomenon can directly lead to dysregulated immune activation and loss of self-tolerance, fostering autoimmunity. In female SLE, over 20 such escape genes (∼10% of expressed X-linked genes have been identified (Silver et al., 2007). Among these, genes such as *CD40LG, CXCR3, IL13RA1, CYBB, IRAK1, TLR7, KDM6A, BTK, CXORF21*, and *TLR8* play crucial roles in the formation of gender dimorphism in SLE (Table 1). These loci bypass XCI in female cells, resulting in elevated immune gene dosage in females relative to males, which an essential mechanism underlying the female-biased incidence of SLE.

**TABLE 1:** Genes that escape from XCI in immune cells.

Gene symbol	Gene nomenclature	Cell type	References
*CD40L*	CD40 ligand	activated T Cells and immortalized B Cell lines generated from pediatric SLE patients or healthy females	Wufuer et al. (2019); Sathishkumar et al. (2018)
*CXCR3*	C-X-C motif chemokine receptor 3		
*IL13RA1*	interleukin 13 receptor subunit alpha 1	pDC from healthy women	Sun et al. (2022)
*CYBB*	cytochrome b-245 beta chain		
*IRAK1*	interleukin 1 receptor associated kinase 1	primary fibroblast cell lines	Chabchoub et al. (2009); Kaufman et al. (2013)
*TLR7*	toll like receptor 7	monocyte, lymphocyte B and pDC from healthy women and Klinefelter syndrome males (XXY)	Santiwatana et al. (2018); Ji et al. (2015)
*KDM6a*	lysine demethylase 6 A	mouse-human somatic cell hybrids	Souyris et al. (2018)
*BTK*	Burton tyrosine kinase	burton tyrosine kinase	Li et al. (2022)
*Cxorf21/TASL*	TLR adaptor interacting with SLC15A4 on the lysosome	pDC from healthy women	Oghumu et al. (2019)
*TLR8*	toll like receptor 8	female monocytes and CD4^+^ T Cells	Harris et al. (2019)

In Graves’ disease (GD), studies have demonstrated variable degrees of XCI skewing in immune cells, with a subset of cells exhibiting severe or extreme skewing (Sauteraud et al., 2021). In other autoimmune diseases such as myasthenia gravis (MG), the frequency of XCI skewing is also significantly higher in female patients than in healthy controls, with this difference being particularly pronounced in younger patients. This observation suggests that XCI skewing may represent a general mechanism contributing to the higher prevalence of autoimmune diseases in females (Zhang et al., 2020).

In recent years, accumulating evidence suggests that XCI escape and XCI skewing do not act in isolation but rather synergistically drive disease progression by co-amplifying the expression of X-linked immune genes, thereby forming a positive feedback loop. Taking *TLR7* as an example, XCI escape enables its biallelic expression in female cells, whereas XCI skewing may cause the X chromosome carrying the risk allele of *TLR7* to predominate in the cell population. Together, these two mechanisms lead to excessive activation of *TLR7* signaling (Scofield et al., 2025). Overactivation of *TLR7* promotes the massive production of IFN-α, which in turn can induce the expression of more XCI escape genes, thereby forming a self-reinforcing vicious cycle. At the epigenetic level, XCI escape genes and XCI skewing-related genes interactively regulate each other. For example, *Kdm6A*, an XCI escape gene, encodes a histone demethylase that modulate chromatin status, thereby affecting the expression of genes involved in XCI skewing. Conversely, XCI skewing may also indirectly regulate the extent of XCI escape by influencing the expression or function of *XIST* (Lovell and Anguera, 2025). In SLE patients, this crosstalk manifests as dysregulation of the overall X-chromosome expression profile and correlates with disease severity (Soares et al., 2025). Single-cell analyses of SLE patients further reveal that XCI escape and XCI skewing are highly co-occurrent in specific immune cell subsets, such as plasma cells and dendritic cells. This suggests that the two mechanisms act synergistically within particular cell types to drive disease progression (Jiwrajka et al., 2023). Moreover, *XIST* RNA itself serves as a ligand for *TLR7*, further potentiating *TLR7* signaling, and XCI skewing may influence *XIST* expression levels, thereby generating synergistic effects with XCI escape at multiple levels (Scofield et al., 2025). Therefore, through interactions at multiple levels including gene expression, epigenetic regulation, and signaling pathways, XCI escape and XCI skewing form a convergent synergistic mechanism that jointly amplifies immune dysregulation in female SLE patients.

In summary, we propose that the synergy between XCI escape and XCI skewing is not a coincidence, but rather a “double-edged sword” bequeathed by evolutionary pressures on the female X chromosome to preserve immune advantage. It confers upon females an enhanced capacity for anti-infection defense, yet, due to the hyperamplification of genes such as *TLR7*, it also renders females more susceptible to autoimmune and neuroimmune dysregulation. A future breakthrough in sex-specific medicine will no longer focus solely on “why females are more susceptible,” but rather on “how to precisely silence this self-amplifying immune loop without compromising anti-infection advantages”.

### 3.2 Neurodevelopmental disorders (NDDS)

Approximately 4% of the X chromosome carries over 20% of genes associated with intellectual disability (Rubin et al., 2024). Notably, neurodevelopment-related escape genes cluster at the telomeric Xp region, which shows enhanced chromatin accessibility during development (Hässler et al., 2022). For example, biallelic expression of the *MECP2* gene can enhance the activity of methyl-CpG binding protein 2, a protein that plays a key role in synaptic plasticity and neuronal maturation. Studies have shown that mutations in *MECP2* are the primary cause of Rett syndrome. Female heterozygous patients exhibit cellular chimerism due to XCI, where cells with normal and mutated *MECP2* coexist, and this chimerism directly influences the severity of the phenotype (Ribeiro and MacDonald, 2020). In neurons, the *KDM5C* gene functions as an XCI escape gene, and its biallelic expression can alter histone demethylase activity, thereby affecting chromatin structure and the gene expression profile. Loss of *KDM5C* function leads to X-linked intellectual disability. Due to *KDM5C* escaping XCI, females typically express higher levels of *KDM5C*, which may explain why male patients are more common and exhibit more severe symptoms (Bonefas et al., 2023). In astrocytes, biallelic expression of the XCI escape gene *SLC6A8* affects creatine transport; mutations in this gene can lead to abnormal energy metabolism, which is associated with neurodevelopmental delay and epilepsy (Li et al., 2024). These findings highlight that the dosage effect of escape genes is a key determinant of central nervous system functional homeostasis and disease susceptibility.

XCI skewing leads to imbalanced expression of specific X-linked genes in female patients with neurodevelopmental disorders, thereby influencing the sex differences in disease phenotypes. A study involving 136 intellectual disability (ID) patients found that extreme XCI skewing can be related to ID phenotypes caused by pathogenic variants in the X-chromosome (Sun et al., 2022). In patients with ASD, biased inactivation of the X chromosome carrying risk alleles of *NLGN3* or *SHANK3* can lead to abnormal expression of synaptic proteins and affect neural circuit formation. For example, in patients with *CDKL5* deficiency disorder, the XCI skewing pattern is closely associated with the disease phenotype. A study reported a female patient carrying a *CDKL5* missense variant who exhibited skewed XCI, and this variant was found to significantly enhance *CDKL5* kinase activity, suggesting that XCI skewing may influence the expression level of the mutant allele (Frasca et al., 2022). This case demonstrates that even among patients carrying the same missense variant, the direction of XCI bias (e.g., whether the mutant allele is preferentially expressed) may directly influence the functional consequences of the mutation (e.g., enhanced kinase activity), thereby determining the severity of the disease phenotype. In individuals with intellectual disability, XCI skewing is associated with CGG repeat expansion in the *FMR1* gene. A study of individuals with idiopathic intellectual disability found that female patients carrying a full *FMR1* mutation exhibit a skewed XCI pattern, with preferential expression of the X chromosome harboring the mutated allele. This may lead to partial expression of the fragile X syndrome (Luo et al., 2021). Furthermore, XCI skewing affects the expression of neuroinflammatory genes such as *CX3CR1*, leading to microglial dysfunction and exacerbating the pathological processes of neurodevelopmental disorders. Studies have shown that the incidence of XCI skewing (>90%) is significantly higher in individuals with undiagnosed neurodevelopmental disorders compared to the general population, suggesting that XCI skewing may mask X-linked pathogenic variants (Giovenino et al., 2023). Findings on XCI skewing in neurodevelopmental disorders reveal a long-overlooked clinical fact: XCI status critically modifies phenotypic expression in females with X-linked disorders. For undiagnosed female NDD patients who test negative for pathogenic variants by routine genetic testing, XCI analysis is warranted, as skewing suggests a potentially concealed X-linked mutation. Accordingly, we advocate integrating XCI testing into the diagnostic pathway for female NDD patients.

XCI escape and XCI skewing jointly regulate synaptic plasticity and neurotransmitter systems, forming a complex regulatory network. For example, biallelic expression of the XCI escape gene *MECP2*, together with abnormal expression of GABA receptor genes (e.g., *GABRA3*) caused by XCI skewing, jointly affects the excitatory-inhibitory balance and is associated with ASD and epilepsy. During critical periods of neurodevelopment, XCI escape and XCI skewing act synergistically by influencing epigenetic reprogramming. The XCI escape gene *KDM5C* can regulate the methylation status of genes associated with XCI skewing, and *vice versa*. As a histone demethylase, loss of *KDM5C* function not only affects chromatin structure but may also interact with XCI skewing by regulating the expression of other X-linked gene (Leonardi et al., 2023). That is to say, *KDM5C* is not only an effector gene of XCI escape but also, through its epigenetic regulatory function, reciprocally influences the expression status of genes related to XCI skewing, thereby forming a “escape-skewing” bidirectional regulatory loop. This mechanism holds important implications for understanding the female protective effect in X-linked neurodevelopmental disorders. Moreover, in NDDS, XCI skewing has been observed in families with mutations in genes escaping XCI, such as *DDX3X*. A female patient with a *de novo DDX3X* mutation exhibited extreme XCI skewing on the mutant allele, with reduced expression of the mutant transcript. This case highlights how familial or *de novo* skewing can influence the phenotypic variability of X-linked disorders (Lukin et al., 2024). These findings indicate that XCI escape and XCI skewing operate with an integrated regulatory framework shaped by epigenetic mechanisms (such as *KDM5C*-mediated histone methylation regulation). This framework further reveals the potential molecular basis of the female protective effect in X-linked NDDS, wherein the dynamic balance between escape and skewing may determine the heterogeneity and severity of clinical symptoms in females.

### 3.3 Cardiovascular diseases (CVD)

Sex chromosomes play a critical role in modulating CVD risk and outcomes, beyond the traditionally recognized influence of sex hormones. This influence is partly mediated by the differential expression of genes that escape X inactivation. These escape genes include chromatin-modifying enzymes that regulate gene expression genome-wide, thereby affecting cardiometabolic traits and contributing to sex differences in CVD susceptibility and progression (Youness et al., 2023). The gene *KDM5C*, which escapes XCI, has been implicated in mediating adverse effects of statin therapy in females, such as new-onset diabetes and muscle weakness. Experimental studies in female mice demonstrated that reducing *KDM5C* expression or supplementing with docosahexaenoic acid (DHA) mitigated statin-induced mitochondrial dysfunction and dysglycemia, effects that parallel observations in women undergoing statin treatment. This highlights how escape genes can modulate drug responses and metabolic pathways critical to cardiovascular health (Moustakli et al., 2025). Female endothelial cells exhibit heightened inflammatory and oxidative stress responses to angiotensin II compared to male cells, partly due to the escape of *NOX2* from XCI, which increases reactive oxygen species production and inflammation, potentially explaining the higher prevalence of certain CVD in women (Zhang et al., 2024). Additionally, the PAR of the X chromosome, which escape inactivation, contain genes whose haploinsufficiency contributes to cardiovascular abnormalities seen in Turner syndrome, such as congenital heart defects and hypertension, further emphasizing the importance of escape genes in cardiovascular pathology (Weber et al., 2023). The longevity-associated variant of the *BPIFB4* gene, expressed from the X chromosome, has also been shown to protect against cardiac ischemia and promote vascularization, suggesting that escape gene variants can confer cardiovascular protection (Fiot et al., 2022). Collectively, escape genes influence CVD risk through diverse mechanisms including modulation of inflammation, mitochondrial function, response to pharmacotherapy, and vascular remodeling. Understanding the roles of these genes offers promising avenues for personalized medicine approaches that account for sex-specific genetic and molecular profiles in CVD management.

XCI skewing also has emerged as a significant factor correlating with CVD risk, particularly in females. A large study analyzing XCI patterns in blood cells from over 1,500 women revealed that XCI skewing correlates with a bias toward myeloid lineage hematopoiesis and an elevated atherosclerotic CVD risk score, which integrates conventional risk factors such as cholesterol levels, blood pressure, and smoking status (Larson et al., 2017). This suggests that XCI skewing reflects underlying changes in hematopoietic stem cell populations that may predispose to vascular pathology. Moreover, clonal hematopoiesis (CH), a condition often linked to XCI skewing, involves somatic mutations in genes like *TET2*, *DNMT3A*, and *ASXL1* that confer a proliferative advantage to mutated hematopoietic clones. CH has been associated with increased incidence of cardiovascular events, likely due to pro-inflammatory states induced by mutant clones, further supporting the link between XCI skewing and cardiovascular risk (Roberts et al., 2022). Beyond risk correlation, XCI skewing appears to play an active role in the progression of CVD. Studies of advanced atherosclerotic plaques in women have demonstrated that nearly half of the plaques exhibit XCI skewing, which is associated with histopathological features indicative of plaque instability, such as plaque hemorrhage (Zitzmann et al., 2025). This suggests that XCI skewing within vascular lesions may influence cellular composition and behavior, potentially exacerbating lesion vulnerability. The presence of XCI skewing in plaques also predicts secondary peripheral artery events within 3 years post-endarterectomy, indicating a prognostic value for disease progression and recurrence (Buono et al., 2023). Taken together, XCI skewing is not merely a biomarker but may actively modulate cellular and molecular pathways that drive CVD progression, offering novel insights into sex-specific mechanisms and potential therapeutic targets.

The role of XCI escape and XCI skewing in causing disease through synergistic regulation of oxidative stress and inflammatory responses in the cardiovascular system has also been extensively documented. For example, biallelic expression of XCI escape genes such as NADPH oxidase 2 (*NOX1*) can increase the production of reactive oxygen species (ROS), while reduced expression of antioxidant genes such as superoxide dismutase 2 (SOD2) resulting from XCI skewing impairs the cellular capacity to scavenge ROS. Together, these factors synergistically exacerbate vascular endothelial injury (Weber et al., 2023). During cardiac remodeling, XCI escape and XCI skewing act synergistically by influencing the TGF-β signaling pathway. Biallelic expression of XCI escape genes such as tissue inhibitor of metalloproteinase 1 (*TIMP1*) suppresses matrix metalloproteinase activity, while aberrant expression of transforming growth factor-beta receptor 2 (*TGFBR2*) resulting from XCI skewing collectively promotes myocardial fibrosis (Spiering et al., 2026). Single-cell transcriptome studies have shown that XCI escape and XCI skewing are highly co-occurring in macrophages and vascular smooth muscle cells within atherosclerotic plaques, and are associated with plaque instability and thrombosis risk (Luo et al., 2023). Collectively, these findings reveal that XCI escape and XCI skewing are not independent or stochastic events, but rather form a synergistic pathogenic mechanism system in cardiovascular disease that spans from oxidative stress to tissue remodeling. They concurrently amplify pro-injury pathways (e.g., *NOX1*-mediated ROS burst) while undermining protective mechanisms (e.g., SOD2 antioxidant capacity and TIMP1/MMP balance), progressively driving the microenvironment toward homeostatic imbalance. Notably, single-cell data confirm the high-frequency co-occurrence of these two mechanisms within specific cell subsets (e.g., macrophages) in atherosclerotic lesions, suggesting that targeting this XCI synergistic interface may open new avenues for plaque stabilization and vascular remodeling intervention.

### 3.4 Cancer

Escape genes from XCI have emerged as critical players in the pathogenesis and progression of female cancers. For example, biallelic expression of the *ATRX* gene can enhance chromatin remodeling activity and inhibit the alternative lengthening of telomeres (ALT) mechanism, a phenomenon closely associated with prognosis in gliomas and pancreatic cancer (Emran et al., 2020). In melanoma, higher expression of X-linked genes that escape XCI, such as *KDM6A* and *ATRX*, is linked to improved survival and enhanced immune infiltration, indicating that X chromosome gene dosage impacts tumor immunity and patient prognosis (Lin et al., 2025). In breast cancer, the histone demethylase *KDM6A*, an escape gene, facilitates the upregulation of the *XIST* lncRNA essential for XCI initiation; loss or mutation of *KDM6A* disrupts XCI and leads to overexpression of oncogenic genes from Xi, contributing to tumorigenesis (Buono et al., 2023). Studies have identified a set of X-linked TSGs with altered promoter methylation and mutational burden in breast cancer, indicating epigenetic dysregulation of escape genes plays a role in oncogenesis (Emran et al., 2020). Transcriptomic analyses reveal that aberrant XCI and escape gene expression patterns correlate with poorer prognosis and more aggressive breast cancer subtypes, such as triple-negative breast cancer (TNBC) (Dhabhai et al., 2022). These findings underscore that escape genes contribute to female cancer biology by influencing gene dosage, epigenetic regulation, tumor suppressor activity, and immune interactions.

XCI skewing plays a multi-level carcinogenic role in the development and progression of cancer. XCI skewing leads to the predominance of a specific parental X chromosome in tumor cells, thereby allowing the expansion of tumor clones harboring the X chromosome that carries oncogenic mutations or inactivated tumor suppressor genes. In ovarian cancer, XCI skewing correlates with the amplification of mutant alleles of *BRCA1* or *BRCA2*, thereby promoting homologous recombination repair deficiency and genomic instability (Liu et al., 2022). The epigenetic landscape shaped by XCI skewing also modulates immune responses in the tumor microenvironment, contributing to sex-specific differences in cancer immunity and therapeutic responses (Rojas et al., 2020; Salama et al., 2020). In patients with acute myeloid leukemia, the degree of XCI skewing is associated with disease relapse and drug resistance. XCI skewing in hematopoietic tissues has been associated with clonal hematopoiesis, a pre-malignant state increasing the risk of hematologic cancers (Larson et al., 2017; Roberts et al., 2022). Furthermore, defects in XCI have been established as an important additional risk factor for cancer in females, accounting for more than 10% of XCI deficiencies and conferring a 40% attributable risk across 12 cancer types (Cáceres et al., 2025). Taken together, these findings demonstrate that XCI skewing serves as a synergistic oncogenic driver that integrates genetic selection, epigenetic regulation, and microenvironment remodeling. Moreover, they underscore the necessity of incorporating XCI status assessment into female cancer risk prediction and personalized therapeutic strategies.

There also exists a synergistic oncogenic mechanism in which XCI escape and XCI skewing collectively regulate gene dosage balance in tumor cells. For example, biallelic expression of the XCI escape gene *ATRX*, together with the amplification of *TP53* mutant alleles resulting from XCI skewing, jointly promotes genomic instability and tumor progression (Caramia et al., 2023). This synergistic effect can be conceptualized as XCI escape providing an initial “dosage license” through biallelic gene expression, while XCI skewing amplifies the “allelic advantage” via clonal selection. Within the tumor immune microenvironment, XCI escape and XCI skewing act synergistically by influencing antigen presentation and immune checkpoint signaling. Biallelic expression of XCI escape genes such as *MAGEC1* enhances tumor antigen expression, while XCI skewing leads to aberrant *PD-L1* expression. Together, these alterations affect immune surveillance and the efficacy of immunotherapy (Zhang et al., 2025). This seemingly paradoxical combination of “increased antigen expression” and “upregulated immune checkpoints” actually constitutes a sophisticated immune evasion strategy. While displaying more antigens, the tumor simultaneously activates stronger immunosuppressive signals, potentially inducing T Cell exhaustion rather than effective clearance. Single-cell analyses have revealed that XCI escape and XCI skewing are highly co-occurrent in cancer stem cell and drug-resistant cell subpopulations across multiple cancer types, including breast, lung, and colorectal cancers, and are significantly associated with patient survival rates (Cáceres et al., 2025). These findings indicate that XCI escape and XCI skewing cooperatively drive cancer progression through multilayered mechanisms involving gene dosage, immune regulation, and clonal evolution. Consequently, targeting the XCI escape/skewing axis may serve as a critical approach to circumvent therapeutic resistance and enhance the efficacy of immunotherapy in female malignancies.

## 4 Application of XCI in cinical research

### 4.1 XCI as biomarkers

**FIGURE 4:**
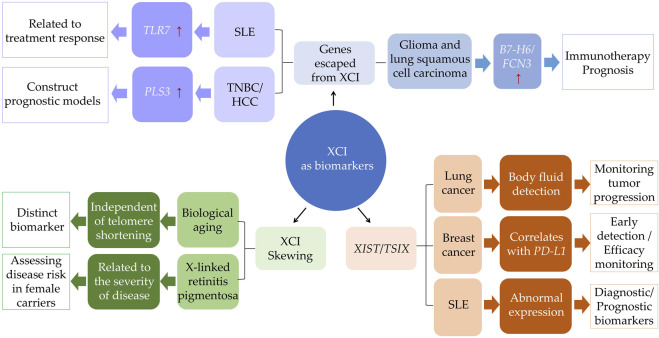
The potential of escape genes from XCI, XCI skewing and *XIST*/*TSIX* as biomarkers in human diseases.

Abnormalities of the XCI pattern, including skewed inactivation and inactivation escape, are closely associated with the occurrence and development of various diseases. Therefore, XCI demonstrates significant value as a biomarker in disease risk prediction, early diagnosis, and personalized treatment. In SLE, the expression of the escape gene *TLR7* is associated with disease activity and treatment response. Moreover, in TNBC and HCC, escape gene-associated signatures have been employed to construct prognostic models for predicting hypoxia, immune evasion, and treatment outcomes (Ba et al., 2025; Yin et al., 2025). Immune checkpoint molecules such as *B7-H6* and *FCN3*, along with the escape gene *PLS3*, are important biomarkers for prognosis and immunotherapy response in multiple tumor types **(** Lin et al., 2023; Mohammadi et al., 2022)**.** XCI skewing is a unique aging biomarker that operates independently of classical aging markers, and its utility in assessing disease risk has been demonstrated in female carriers of retinitis pigmentosa (Larson et al., 2017; Li et al., 2025). *XIST* serves as a biomarker for some diseases, supporting early intervention and non-invasive monitoring. In lung cancer, its levels reflect tumor progression and treatment response (Peeters et al., 2023). In breast cancer, the expression of *XIST* and *TSIX* correlates with *PD-L1* status and exhibits subtype-specific differences, enabling early detection and treatment efficacy monitoring (Emran et al., 2020). In systemic lupus erythematosus, aberrant *XIST* expression holds diagnostic and prognostic value. Animal studies have demonstrated that modulating Xist levels can suppress disease progression, highlighting its therapeutic potential (Peeters et al., 2023) (Figure 4).

However, the clinical application of XCI as biomarkers faces three core challenges: robustness, variability, and clinical feasibility. The robustness of XCI is fundamental to its utility as a reliable biomarker. Existing studies reveal significant inconsistencies in XCI under different testing conditions, primarily constrained by detection method, locus, and tissue. For instance, the HUMARA assay has a 10%–20% rate of non-informative results and may fail to reflect true XCI status (Fadra et al., 2024). Second, a single detection locus such as the AR gene cannot represent the inactivation status of the entire X chromosome. Finally, the degree of XCI skewing varies considerably across different tissues, as evidenced by distinct results obtained from blood, buccal mucosa, and skin samples (Wagenhäuser et al., 2022).

The variability of XCI is another major barrier to its clinical application, with this variability manifesting between individuals, between tissues, and over time. For example, a study of 248 healthy females demonstrated significant inter-individual differences in the expression of XCI escape genes (Zito et al., 2023). Another study found that XCI ratios are highly consistent between the liver and whole blood, but exhibit greater variability in brain tissue, reflecting differences in cell numbers during lineage specification in embryonic development (Werner et al., 2022).

The clinical feasibility of XCI is limited by detection costs, technical barriers, and insufficient standardization. Traditional AR testing is low in cost but suffers from missing information and inconsistent results. Emerging sequencing methods can address some of these limitations, yet their high cost and complex analytical requirements hinder widespread adoption. The lack of unified standards across laboratories in methods, analysis, and threshold definitions makes cross-study comparisons difficult. Therefore, advancing standardization, reducing costs, and streamlining analytical workflows are key to improving the clinical feasibility of XCI.

In summary, the clinical translation of XCI biomarkers represents a systematic project transitioning from “technological exploration” to “clinical implementation”. Only through standardized detection, multi-omics integration, cost optimization, and AI empowerment can the barriers to their robustness, variability, and feasibility be gradually eliminated.

### 4.2 XCI-based therapeutic strategies for X-linked disorders

The development of novel therapeutic strategies based on the mechanisms of XCI presents a promising frontier in the treatment of various X-linked genetic diseases. In X-linked disorders, targeted reactivation of the silenced wild-type alleles on the Xi represents a promising therapeutic approach for X-linked disorders.

XCI factors (XCIFs) play essential roles in the selective silencing of X-linked genes. Recent studies have identified two such factors, activin A receptor type I (ACVR1) and 3-phosphoinositide-dependent protein kinase 1 (PDPK1). ACVR1 is proposed to sustain Xist expression by regulating its transcription and local chromatin state, whereas PDPK1 facilitates gene reactivation by influencing *Xist* RNA localization, stability, and chromatin accessibility. Inhibition of ACVR1 and PDPK1 in female mouse brain cells resulted in reactivation of the Xi-linked gene methyl-CpG-binding protein 2 (Mecp2). Combined treatment with ACVR1 and PDPK1 inhibitors achieved approximately 30% reactivation of Xi-linked Mecp2-GFP reporter in female cortical neurons (Kim et al., 2025). However, small-molecule inhibitors may broadly reactivate X-linked genes rather than selectively targeting specific loci (e.g., *MECP2*), potentially leading to genomic dosage imbalance. Therefore, optimizing inhibitors to selectively target factors involved in XCI is essential to minimize off-target effects and ensure therapeutic safety.

*XIST*-targeting antisense oligonucleotides (ASOs), such as gapmers1, binds to *XIST* RNA, utilizing the RNA degradation mechanism mediated by ribonuclease H (RNase H) to selectively degrade *XIST* RNA, thereby promoting the activation of the *MECP2* gene on the Xi. Combined inhibition of DNA methylation—using agents such as 5-aza-2-deoxycytidine (Aza)—and *XIST*-directed ASOs treatment has emerged as a promising strategy for reactivating genes on the Xi. In mouse embryonic fibroblasts (MEFs) with a *MECP2*-luciferase reporter, this combined treatment results in a 2%–5% restoration of normal *Mecp2* levels (Przanowski et al., 2018). In the context of diseases like Fabry disease, where XCI leads to variable expression of the α-galactosidase A enzyme, ASOs could be designed to reactivate the silent allele on the inactive X chromosome, thereby restoring enzyme function and ameliorating disease symptoms. Nevertheless, Aza is not suitable for chronic neurodegenerative diseases due to its systemic toxicity (e.g., myelosuppression), and the necessity of long-term repeated treatment remains unclear. Antisense oligonucleotides (ASOs) targeting *XIST* carry risks of off-target effects, whereas advanced delivery strategies such as lipid nanoparticles may improve their selectivity and safety.

Targeting the escape characteristics of X-linked immune-related genes such as *TLR7*, *TLR8*, and *BTK*, small molecule inhibitors can be designed to selectively modulate female-specific immune response pathways. Moreover, the exploration of small molecule inhibitors targeting epigenetic regulators such as EZH2, a key player in the maintenance of XCI, has emerged as a potential strategy in cancer therapy. Inhibition of EZH2 has been shown to disrupt the silencing of TSGs on the X chromosome, thereby promoting their expression and potentially enhancing anti-tumor responses (Carrette et al., 2018).

**FIGURE 5:**
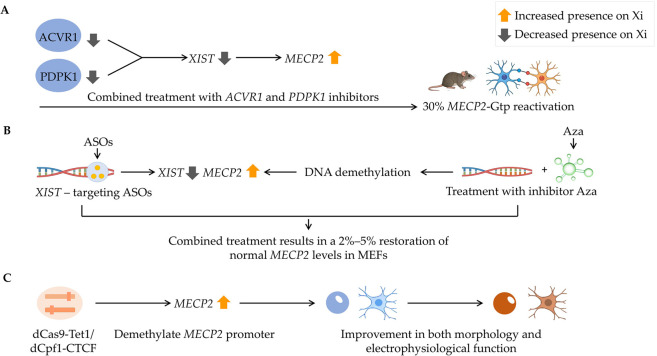
The inactive X chromosome (Xi) reactivation as a therapeutic approach for X-linked disorders. **(A)** Reactivation of *MECP2* in female mouse brain cells using ACVR1 and PDPK1 inhibitors. **(B)** Synergistic reactivation of the X-inactive chromosome via DNA demethylation and *XIST*-targeting antisense oligonucleotides. **(C)** Reactivation of *MECP2* using dCas9 and dCpf1 editors.

In addition to drug inhibition, gene editing technologies, such as CRISPR/Cas9, could be employed to selectively reactivate genes that escape inactivation or to correct the underlying genetic anomalies that contribute to XCI skewing. Recent studies have used dCas9-Tet1 and dCpf1-CTCF tools to demethylate the *MECP2* promoter, successfully reactivating Xi-linked *MECP2* in RTT human embryonic stem cells. The derived neurons showed improvement in both morphology and electrophysiological function, indicating functional recovery (Cheng et al., 2024). Furthermore, the reactivation of silent wild-type alleles was achieved in XLSA by using Aza and in brain iron accumulation disease through biotin supplementation (Morimoto et al., 2022; Qian et al., 2023) (Figure 5). Although gene editing and epigenetic reprogramming technologies are encouraging, several key issues including targeting specificity, delivery efficiency, long-term safety, and interindividual heterogeneity must be addressed before their clinical application.

These findings illustrate the dual-edged nature of Xi reactivation strategies. Targeted reactivation of specific genes can correct disease-associated haploinsufficiency. However, global Xi reactivation risks aberrant expression of oncogenes or immune-related genes, potentially leading to malignant progression or autoimmune pathology.

## 5 Future research directions

Despite significant advances in understanding XCI and its implications for human diseases, critical questions remain unresolved. A fundamental question is that the interplay between local regulatory sequences of XCI and the broader chromatin domain context remains poorly understood and requires elucidation. Another question is how epigenetic modifications dynamically regulate escape status during development and disease states. Future mechanistic studies should also integrate the analysis of XCI skewing patterns with the functional characterization of escape genes. Key questions to be addressed include how imbalanced expression of escape genes directly drives skewing-associated clonal expansion, either by conferring a growth advantage or by inducing functional deficiencies, and how altered escape gene expression acts as an upstream event that influences cellular function, disrupts signaling pathways, and ultimately increases disease susceptibility. The overarching goal is to elucidate the molecular mechanisms through which these two factors synergistically shape cell fate and organismal homeostasis.

Future breakthroughs in this field depend on the synergistic advancement of technologies, models, and translational research. At the basic research level, there is an urgent need to develop single-cell tools capable of simultaneously resolving X chromosome activity status, clonal lineage, epigenome, and transcriptome, enabling the reconstruction of the dynamic evolutionary trajectory of XCI at single-cell resolution. Regarding mechanistic exploration, efforts should focus on utilizing organoid or *in vivo* models to dissect how specific escape genes influence cell selection through dosage effects. At the clinical translation level, enhancing XCI as a biomarker requires multidisciplinary integration. Strategies include combining XCI methylation with transcriptomic and proteomic profiles to improve robustness, developing rapid digital PCR-based kits for *XIST* detection to reduce technical barriers, applying AI and machine learning to identify disease-specific patterns and build predictive models, and conducting multicenter prospective studies to generate high-level evidence. Ultimately, translational research should continue exploring strategies to moderately correct pathological XCI skewing or modulate key escape gene expression using small-molecule drugs or gene editing technologies, with the aim of delaying tissue aging or treating related diseases. Developing site-specific epigenetic interventions, rather than global *XIST* silencing or erasure, will be essential to ensure the safety of therapeutic strategies.

## References

[B1] Aguado B. A. Walker C. J. Grim J. C. Schroeder M. E. Batan D. Vogt B. J. (2022). Genes that escape X chromosome inactivation modulate sex differences in valve myofibroblasts. Circulation 145, 513–530. 10.1161/CIRCULATIONAHA.121.054108 35000411 PMC8844107

[B2] Ayachi S. Buscarlet M. Busque L. (2020). 60 years of clonal hematopoiesis research: from X-chromosome inactivation studies to the identification of driver mutations. Exp. Hematol. 83, 2–11. 10.1016/j.exphem.2020.01.008 32001340

[B3] Ba L. Zhao Z. Zhang C. Chu Y. Wu C. (2025). Expression and prognostic impact of hypoxia- and immune escape-related genes in triple-negative breast cancer: a comprehensive analysis. Int. Immunopharmacol. 146, 113810. 10.1016/j.intimp.2024.113810 39689602

[B4] Balaton B. P. Brown C. J. (2021). Contribution of genetic and epigenetic changes to escape from X-chromosome inactivation. Epigenet. Chromatin. 14, 30. 10.1186/s13072-021-00404-9 PMC8244145 34187555

[B5] Balaton B. P. Fornes O. Wasserman W. W. Brown C. J. (2021). Cross-species examination of X-chromosome inactivation highlights domains of escape from silencing. Epigenet. Chromatin. 14, 12. 10.1186/s13072-021-00386-8 PMC7890635 33597016

[B6] Bansal P. Kondaveeti Y. Pinter S. F. (2019). Forged by DXZ4, FIRRE, and ICCE: how tandem repeats shape the active and inactive x chromosome. Front. Cell Dev. Biol. 7, 328. 10.3389/fcell.2019.00328 32076600 PMC6985041

[B7] Barakat T. S. Gunhanlar N. Pardo C. G. Achame E. M. Ghazvini M. Boers R. (2011). RNF12 activates Xist and is essential for X chromosome inactivation. PLoS Genet. 7, e1002001. 10.1371/journal.pgen.1002001 21298085 PMC3029249

[B8] Berletch J. B. Yang F. Disteche C. M. (2010). Escape from X inactivation in mice and humans. Genome Biol. 11, 213. 10.1186/gb-2010-11-6-213 20573260 PMC2911101

[B9] Bhangu K. S. Yang C. Illing H. J. Brown C. J. (2025). Impact of somatic XIST deletions on ongoing XIST expression and inactive X silencing and heterochromatin. Hum. Mol. Genet. 34, 2027–2041. 10.1093/hmg/ddaf160 41128597 PMC12681265

[B10] Bonefas K. M. Vallianatos C. N. Raines B. Tronson N. C. Iwase S. (2023). Sexually dimorphic alterations in the transcriptome and behavior with loss of histone demethylase *KDM5C* . Cells 12, 637. 10.3390/cells12040637 36831303 PMC9954040

[B11] Brown C. J. Hendrich B. D. Rupert J. L. Lafrenière R. G. Xing Y. Lawrence J. (1992). The human XIST gene: analysis of a 17 Kb inactive X-specific RNA that contains conserved repeats and is highly localized within the nucleus. Cell 71, 527–542. 10.1016/0092-8674(92)90520-m 1423611

[B12] Buono M. F. Benavente E. D. Daniels M. Mol B. M. Mekke J. M. de Borst G. J. (2023). X chromosome inactivation skewing is common in advanced carotid atherosclerotic lesions in females and predicts secondary peripheral artery events. Biol. Sex. Differ. 14, 43. 10.1186/s13293-023-00527-6 37408072 PMC10324263

[B13] Cáceres A. Pérez-Jurado L. A. Alegret-García A. Dwaraka V. B. Smith R. González J. R. (2025). Defective X-chromosome inactivation and cancer risk in women. Commun. Biol. 8, 289. 10.1038/s42003-025-07691-y 39987288 PMC11846847

[B14] Cantone I. Fisher A. G. (2017). Human X chromosome inactivation and reactivation: implications for cell reprogramming and disease. Philos. Trans. R. Soc. Lond. B. Biol. Sci. 372, 20160358. 10.1098/rstb.2016.0358 28947657 PMC5627160

[B15] Caramia F. Speed T. P. Shen H. Haupt Y. Haupt S. (2023). Establishing the link between X-Chromosome aberrations and *TP53* status, with breast cancer patient outcomes. Cells 12, 2245. 10.3390/cells12182245 37759468 PMC10526523

[B16] Carrette L. L. G. Wang C. Y. Wei C. Press W. Ma W. Kelleher R. J. (2018). A mixed modality approach towards Xi reactivation for Rett syndrome and other X-linked disorders. Proc. Natl. Acad. Sci. USA. 115, E668–E675. 10.1073/pnas.1715124115 29282321 PMC5789928

[B17] Chabchoub G. Uz E. Maalej A. Mustafa C. A. Rebai A. Mnif M. (2009). Analysis of skewed X-chromosome inactivation in females with rheumatoid arthritis and autoimmune thyroid diseases. Arthritis Res. Ther. 11, R106. 10.1186/ar2759 19589151 PMC2745787

[B18] Chen W. Xu J. Zeng G. Ou R. Yang C. Xu C. (2025). Whole-genome profiling of age- and sex-associated DNA methylation signatures in human plasma cell-free DNA. Commun. Med. (Lond). 5, 503. 10.1038/s43856-025-01220-y 41315810 PMC12663547

[B19] Cheng Y. Song Z. Fang X. Tang Z. (2024). Polycomb repressive complex 2 and its core component EZH2: potential targeted therapeutic strategies for head and neck squamous cell carcinoma. Clin. Epigenet. 16, 54. 10.1186/s13148-024-01666-2 PMC11007890 38600608

[B20] Chlamydas S. Markouli M. Strepkos D. Piperi C. (2022). Epigenetic mechanisms regulate sex-specific bias in disease manifestations. J. Mol. Med. 100, 1111–1123. 10.1007/s00109-022-02227-x 35764820 PMC9244100

[B21] Chureau C. Chantalat S. Romito A. Galvani A. Duret L. Avner P. (2011). Ftx is a non-coding RNA which affects xist expression and chromatin structure within the X-inactivation center region. Hum. Mol. Genet. 20, 705–718. 10.1093/hmg/ddq516 21118898

[B22] Coker H. Wei G. Moindrot B. Mohammed S. Nesterova T. Brockdorff N. (2020). The role of the xist 5’ m6A region and RBM15 in X chromosome inactivation. Wellcome Open Res. 5, 31. 10.12688/wellcomeopenres.15711.1 32258426 PMC7097882

[B23] Costanzi C. Pehrson J. R. (1998). Histone macroH2A1 is concentrated in the inactive X chromosome of female mammals. Nature 393, 599–601. 10.1038/31275 9634239

[B24] Costanzi C. Stein P. Worrad D. M. Schultz R. M. Pehrson J. R. (2000). Histone macroH2A1 is concentrated in the inactive X chromosome of female preimplantation mouse embryos. Development 127, 2283–2289. 10.1242/dev.127.11.2283 10804171

[B25] Dardik R. Avishai E. Lalezari S. Barg A. A. Levy-Mendelovich S. Budnik I. (2021). Molecular mechanisms of skewed X-chromosome inactivation in female hemophilia patients-lessons from wide genome analyses. Int. J. Mol. Sci. 22, 9074. 10.3390/ijms22169074 34445777 PMC8396640

[B26] de Oliveira L. A. Piergiorge R. M. Santos-Rebouças C. B. (2025). Escape genes from X-chromosome inactivation: new insights into candidate genes for intellectual disability in females. World J. Biol. Psychiatry. 26, 255–266. 10.1080/15622975.2025.2517040 40556335

[B27] Dhabhai B. Sharma A. Maciaczyk J. Dakal T. C. (2022). X-linked tumor suppressor genes act as presumed contributors in the sex chromosome-autosome crosstalk in cancers. Cancer Invest. 40, 103–110. 10.1080/07357907.2021.1981364 34519229

[B28] Di Giosaffatte N. Valiante M. Tricarico S. Parise G. De Negri A. M. Ricciotti G. (2022). A novel hypothesis on choroideremia-manifesting female carriers: could CHM in-frame variants exert a dominant negative effect? A case report. Genes 13, 1268. 10.3390/genes13071268 35886051 PMC9321261

[B29] Disteche C. M. Berletch J. B. (2015). X-chromosome inactivation and escape. J. Genet. 94, 591–599. 10.1007/s12041-015-0574-1 26690513 PMC4826282

[B30] Dossin F. Heard E. (2022). The molecular and nuclear dynamics of X-chromosome inactivation. Cold Spring Harb. Perspect. Biol. 14, a040196. 10.1101/cshperspect.a040196 34312245 PMC9121902

[B31] Dossin F. Pinheiro I. Żylicz J. J. Roensch J. Collombet S. Le Saux A. (2020). SPEN integrates transcriptional and epigenetic control of X-inactivation. Nature 578, 455–460. 10.1038/s41586-020-1974-9 32025035 PMC7035112

[B32] Emran A. A. Nsengimana J. Punnia-Moorthy G. Schmitz U. Gallagher S. J. Newton-Bishop J. (2020). Study of the female sex survival advantage in melanoma-a focus on X-linked epigenetic regulators and immune responses in two cohorts. Cancers 12, 2082. 10.3390/cancers12082082 32731355 PMC7464825

[B33] Errigo A. Bitti A. Galistu F. Salis R. Pes G. M. Dore M. P. (2023). Relationship between glucose-6-phosphate dehydrogenase deficiency, X-chromosome inactivation and inflammatory markers. Antioxidants (Basel) 12, 334. 10.3390/antiox12020334 36829893 PMC9952105

[B34] Fadra N. Schultz-Rogers L. E. Chanana P. Cousin M. A. Macke E. L. Ferrer A. (2024). Identification of skewed X chromosome inactivation using exome and transcriptome sequencing in patients with suspected rare genetic disease. BMC Genomics 25, 371. 10.1186/s12864-024-10240-2 38627676 PMC11020449

[B35] Fang H. Disteche C. M. Berletch J. B. (2019). X inactivation and escape: epigenetic and structural features. Front. Cell Dev. Biol. 7, 219. 10.3389/fcell.2019.00219 31632970 PMC6779695

[B36] Fang H. Tronco A. R. Bonora G. Nguyen T. Thakur J. Berletch J. B. (2025). CTCF-Mediated insulation and chromatin environment modulate car5b escape from X inactivation. BMC Biol. 23, 68. 10.1186/s12915-025-02137-7 40025499 PMC11874400

[B37] Fiot E. Alauze B. Donadille B. Samara-Boustani D. Houang M. De Filippo G. (2022). Turner syndrome: french national diagnosis and care rotocol (NDCP; national diagnosis and care protocol). Orphanet J. Rare Dis. 17, 261. 10.1186/s13023-022-02423-5 35821070 PMC9277788

[B38] Frasca A. Pavlidou E. Bizzotto M. Gao Y. Balestra D. Pinotti M. (2022). Not just loss-of-function variations: identification of a hypermorphic variant in a patient with a *CDKL5* missense substitution. Neurol. Genet. 8, e666. 10.1212/NXG.0000000000000666 35280940 PMC8906656

[B39] Gendrel A. V. Apedaile A. Coker H. Termanis A. Zvetkova I. Godwin J. (2012). Smchd1-dependent and -independent pathways determine developmental dynamics of CpG island methylation on the inactive X chromosome. Dev. Cell 23, 265–279. 10.1016/j.devcel.2012.06.011 22841499 PMC3437444

[B40] Giovenino C. Trajkova S. Pavinato L. Cardaropoli S. Pullano V. Ferrero E. (2023). Skewed X-chromosome inactivation in unsolved neurodevelopmental disease cases can guide re-evaluation for X-linked genes. Eur. J. Hum. Genet. 31, 1228–1236. 10.1038/s41431-023-01324-w 36879111 PMC10620389

[B41] Grigoryan A. Pospiech J. Krämer S. Lipka D. Liehr T. Geiger H. (2021). Attrition of X chromosome inactivation in aged hematopoietic stem cells. Stem Cell Rep. 16, 708–716. 10.1016/j.stemcr.2021.03.007 PMC8072063 33798450

[B42] Harris V. M. Koelsch K. A. Kurien B. T. Harley I. T. W. Wren J. D. Harley J. B. (2019). Characterization of Cxorf21 provides molecular insight into female-bias immune response in SLE pathogenesis. Front. Immunol. 10, 2160. 10.3389/fimmu.2019.02160 31695690 PMC6816314

[B43] Hässler S. Camilleri-Broët S. Allez M. Deisenhammer F. Fogdell-Hahn A. Mariette X. (2022). A genetic association test accounting for skewed X-inactivation with application to biotherapy immunogenicity in patients with autoimmune diseases. Front. Med. 9, 856917. 10.3389/fmed.2022.856917 PMC9199462 35721087

[B44] Heard E. Rougeulle C. Arnaud D. Avner P. Allis C. D. Spector D. L. (2001). Methylation of histone H3 at lys-9 is an early mark on the X chromosome during X inactivation. Cell 107, 727–738. 10.1016/s0092-8674(01)00598-0 11747809

[B45] Hoelzl S. Hasenbein T. P. Engelhardt S. Andergassen D. (2025). Aging promotes reactivation of the barr body at distal chromosome regions. Nat. Aging. 5, 984–996. 10.1038/s43587-025-00856-8 40312568 PMC12176624

[B46] Huang Y. Ma L. Zhang Z. Nie S. Zhou Y. Zhang J. (2023). Nance-horan syndrome pedigree due to a novel microdeletion and skewed X chromosome inactivation. Mol. Genet. Genomic Med. 11, e2100. 10.1002/mgg3.2100 36370055 PMC9938751

[B47] Jachowicz J. W. Strehle M. Banerjee A. K. Blanco M. R. Thai J. Guttman M. (2022). Xist spatially amplifies SHARP/SPEN recruitment to balance chromosome-wide silencing and specificity to the X chromosome. Nat. Struct. Mol. Biol. 29, 239–249. 10.1038/s41594-022-00739-1 35301492 PMC8969943

[B48] Ji B. Higa K. K. Kelsoe J. R. Zhou X. (2015). Over-expression of XIST, the master gene for X chromosome inactivation, in females with major affective disorders. EBioMedicine 2, 909–918. 10.1016/j.ebiom.2015.06.012 26425698 PMC4563114

[B49] Jiwrajka N. Toothacre N. E. Beethem Z. T. Sting S. Forsyth K. S. Dubin A. H. (2023). Impaired dynamic X-chromosome inactivation maintenance in T cells is a feature of spontaneous murine SLE that is exacerbated in female-biased models. J. Autoimmun. 139, 103084. 10.1016/j.jaut.2023.103084 37399593 PMC11140471

[B50] Kaufman K. M. Zhao J. Kelly J. A. Hughes T. Adler A. Sanchez E. (2013). Fine mapping of Xq28: both MECP2 and IRAK1 contribute to risk for systemic lupus erythematosus in multiple ancestral groups. Ann. Rheum. Dis. 72, 437–444. 10.1136/annrheumdis-2012-201851 22904263 PMC3567234

[B51] Kawashima S. Hattori A. Suzuki E. Matsubara K. Toki M. Kosaki R. (2021). Methylation status of genes escaping from X-chromosome inactivation in patients with X-chromosome rearrangements. Clin. Epigenet. 13, 134. 10.1186/s13148-021-01121-6 PMC8244138 34193245

[B52] Kim Y. R. Jung Y. Kang I. Yeo E. J. (2025). Understanding sex differences in autoimmune diseases: immunologic mechanisms. Int. J. Mol. Sci. 26, 7101. 10.3390/ijms26157101 40806232 PMC12346812

[B53] Korotkova D. G. Karpova M. I. Galiulina K. Y. Vasilenko A. F. Shestakova M. V. Kashko T. N. (2025). A case of becker muscular dystrophy in a woman with skewed X-chromosome inactivation. Zh. Nevrol. Psikhiatr. Im. S. S. Korsakova 125, 139–144. 10.17116/jnevro2025125011139 39930689

[B54] Larson N. B. Fogarty Z. C. Larson M. C. Kalli K. R. Lawrenson K. Gayther S. (2017). An integrative approach to assess X-chromosome inactivation using allele-specific expression with applications to epithelial ovarian cancer. Genet. Epidemiol. 41, 898–914. 10.1002/gepi.22091 29119601 PMC5726546

[B55] Leonardi E. Aspromonte M. C. Drongitis D. Bettella E. Verrillo L. Polli R. (2023). Expanding the genetics and phenotypic spectrum of Lysine-specific demethylase 5C (KDM5C): a report of 13 novel variants. Eur. J. Hum. Genet. 31, 202–215. 10.1038/s41431-022-01233-4 36434256 PMC9905063

[B56] Li S. Lund J. B. Christensen K. Baumbach J. Mengel-From J. Kruse T. (2020). Exploratory analysis of age and sex dependent DNA methylation patterns on the X-chromosome in whole blood samples. Genome Med. 12, 39. 10.1186/s13073-020-00736-3 32345361 PMC7189689

[B57] Li J. Ming Z. Yang L. Wang T. Liu G. Ma Q. (2022). Long noncoding RNA XIST: mechanisms for X chromosome inactivation, roles in sex-biased diseases, and therapeutic opportunities. Genes Dis. 9, 1478–1492. 10.1016/j.gendis.2022.04.007 36157489 PMC9485286

[B58] Li Y. Qin L. Yang K. Chen X. Zhu H. Mi L. (2024). Clinical features and genetic analysis of 17 Chinese pedigrees affected with X-linked intellectual disability. Zhonghua Yi Xue Yi Chuan Xue Za Zhi 41, 533–539. 10.3760/cma.j.cn511374-20230808-00048 38684296

[B59] Li W. Zu L. Xu S. (2025). FCN3 can serve as a potential biomarker for prognosis and immunotherapy of lung squamous cell carcinoma. Chin. J. Lung Cancer 28, 114–130. 10.3779/j.issn.1009-3419.2025.105.01 PMC11931238 40114488

[B60] Lin S. Li J. Zhao R. Yu M. Peng L. (2023). Oxeiptosis core genes and their multi-omics analysis in hepatocellular carcinoma. Med. Baltim. 102, e36051. 10.1097/MD.0000000000036051 PMC10637422 37960791

[B61] Lin J. Zhang J. Ma L. Fang H. Ma R. Groneck C. (2025). KDM6A facilitates xist upregulation at the onset of X inactivation. Biol. Sex. Differ. 16, 1. 10.1186/s13293-024-00683-3 39754175 PMC11699772

[B62] Liu F. Han Q. Zhang T. Chang F. Deng J. Huang X. (2022). CRL4-DCAF8L1 regulates BRCA1 and BARD1 protein stability. Int. J. Biol. Sci. 18, 1434–1450. 10.7150/ijbs.57178 35280675 PMC8898372

[B63] Lovell C. D. Anguera M. C. (2025). More x’s, more problems: how contributions from the X chromosomes enhance female predisposition for autoimmunity. Curr. Opin. Immunol. 93, 102543. 10.1016/j.coi.2025.102543 40020257 PMC11909602

[B64] Lukin J. Smith C. M. De Rubeis S. (2024). Emerging X-linked genes associated with neurodevelopmental disorders in females. Curr. Opin. Neurobiol. 88, 102902. 10.1016/j.conb.2024.102902 39167997 PMC11392613

[B65] Luo S. He W. Liao Y. Tang W. Li X. Hu L. (2021). Analysis and prenatal diagnosis of FMR1 gene mutations among patients with unexplained mental retardation. Zhonghua Yi Xue Yi Chuan Xue Za Zhi 38, 439–445. 10.3760/cma.j.cn511374-20200513-00344 33974251

[B66] Luo L. Fu C. Bell C. F. Wang Y. Leeper N. J. (2023). Role of vascular smooth muscle cell clonality in atherosclerosis. Front. Cardiovasc Med. 10, 1273596. 10.3389/fcvm.2023.1273596 38089777 PMC10713728

[B67] Lyon M. F. (1961). Gene action in the X-chromosome of the mouse (mus musculus L.). Nature 190, 372–373. 10.1038/190372a0 13764598

[B68] McHugh C. A. Chen C. K. Chow A. Surka C. F. Tran C. McDonel P. (2015). The xist lncRNA interacts directly with SHARP to silence transcription through HDAC3. Nature 521, 232–236. 10.1038/nature14443 25915022 PMC4516396

[B69] Migeon B. R. Haisley-Royster C. (1998). Familial skewed X inactivation and X-linked mutations: unbalanced X inactivation is a powerful means to ascertain X-linked genes that affect cell proliferation. Am. J. Hum. Genet. 62, 1555–1557. 10.1086/301858 9585586 PMC1377137

[B122] Minks J. Robinson W. P. Brown C. J. (2008). A skewed view of X chromosome inactivation. J. Clin. Invest. 118, 20–23. 10.1172/JCI34470 18097476 PMC2147673

[B70] Mohammadi A. Najafi S. Amini M. Mansoori B. Baghbanzadeh A. Hoheisel J. D. (2022). The potential of B7-H6 as a therapeutic target in cancer immunotherapy. Life Sci. 304, 120709. 10.1016/j.lfs.2022.120709 35697295

[B71] Morimoto Y. Chonabayashi K. Kawabata H. Okubo C. Yamasaki-Morita M. Nishikawa M. (2022). Azacitidine is a potential therapeutic drug for pyridoxine-refractory female X-linked sideroblastic anemia. Blood Adv. 6, 1100–1114. 10.1182/bloodadvances.2021005664 34781359 PMC8864662

[B72] Moustakli E. Potiris A. Zikopoulos A. Mavrogianni D. Kathopoulis N. Drakaki E. (2025). Sex differences in hypertension risk: insights from placental genomics and pregnancy-driven vascular programming. Int. J. Mol. Sci. 26, 6034. 10.3390/ijms26136034 40649813 PMC12250033

[B73] Naik H. C. Hari K. Chandel D. Jolly M. K. Gayen S. (2022). Single-cell analysis reveals X upregulation is not global in pre-gastrulation embryos. iScience 25, 104465. 10.1016/j.isci.2022.104465 35707719 PMC9189126

[B74] Oghumu S. Varikuti S. Stock J. C. Volpedo G. Saljoughian N. Terrazas C. A. (2019). Cutting edge: CXCR3 escapes X chromosome inactivation in T cells during infection: potential implications for sex differences in immune responses. J. Immunol. 203, 789–794. 10.4049/jimmunol.1800931 31253729 PMC6684832

[B75] Peeters S. B. Leung T. Fornes O. Farkas R. A. Wasserman W. W. Brown C. J. (2023). Refining the genomic determinants underlying escape from X-chromosome inactivation. Nar. Genomics Bioinf 5, lqad052. 10.1093/nargab/lqad052 PMC10227363 37260510

[B76] Plath K. Fang J. Mlynarczyk-Evans S. K. Cao R. Worringer K. A. Wang H. (2003). Role of histone H3 lysine 27 methylation in X inactivation. Science 300, 131–135. 10.1126/science.1084274 12649488

[B77] Plath K. Talbot D. Hamer K. M. Otte A. P. Yang T. P. Jaenisch R. (2004). Developmentally regulated alterations in polycomb repressive complex 1 proteins on the inactive X chromosome. J. Cell Biol. 167, 1025–1035. 10.1083/jcb.200409026 15596546 PMC2172612

[B78] Przanowski P. Wasko U. Zheng Z. Yu J. Sherman R. Zhu L. J. (2018). Pharmacological reactivation of inactive X-linked Mecp2 in cerebral cortical neurons of living mice. Proc. Natl. Acad. Sci. USA. 115, 7991–7996. 10.1073/pnas.1803792115 30012595 PMC6077728

[B79] Pyfrom S. Paneru B. Knox J. J. Cancro M. P. Posso S. Buckner J. H. (2021). The dynamic epigenetic regulation of the inactive X chromosome in healthy human B cells is dysregulated in lupus patients. Proc. Natl. Acad. Sci. U. S. A. 118, e2024624118. 10.1073/pnas.2024624118 34103397 PMC8214693

[B80] Qian J. Guan X. Xie B. Xu C. Niu J. Tang X. (2023). Multiplex epigenome editing of MECP2 to rescue Rett syndrome neurons. Sci. Transl. Med. 15, eadd4666. 10.1126/scitranslmed.add4666 36652535 PMC11975455

[B81] Raposo A. C. Caldas P. Jeremias J. Arez M. Cazaux Mateus F. Barbosa P. (2025). Gene reactivation upon erosion of X chromosome inactivation in female hiPSCs is predictable yet variable and persists through differentiation. Stem Cell Rep. 20, 102472. 10.1016/j.stemcr.2025.102472 PMC12143139 40185090

[B82] Ravid Lustig L. Sampath Kumar A. Schwämmle T. Dunkel I. Noviello G. Limberg E. (2023). GATA transcription factors drive initial xist upregulation after fertilization through direct activation of long-range enhancers. Nat. Cell Biol. 25, 1704–1715. 10.1038/s41556-023-01266-x 37932452 PMC10635832

[B83] Ribeiro M. C. MacDonald J. L. (2020). Sex differences in *Mecp2*-mutant Rett syndrome model mice and the impact of cellular mosaicism in phenotype development. Brain Res. 1729, 146644. 10.1016/j.brainres.2019.146644 31904347 PMC7024565

[B84] Roberts A. L. Morea A. Amar A. Zito A. El-Sayed Moustafa J. S. Tomlinson M. (2022). Age acquired skewed X chromosome inactivation is associated with adverse health outcomes in humans. Elife 11, e78263. 10.7554/eLife.78263 36412098 PMC9681199

[B85] Roberts A. L. Morea A. Amar A. West M. Karrar S. Lehane R. (2024). Haematopoietic stem cell-derived immune cells have reduced X chromosome inactivation skewing in systemic lupus erythematosus. Ann. Rheum. Dis. 83, 1315–1321. 10.1136/ard-2024-225585 38937070 PMC11503196

[B86] Rodermund L. Coker H. Oldenkamp R. Wei G. Bowness J. Rajkumar B. (2021). Time-resolved structured illumination microscopy reveals key principles of xist RNA spreading. Science 372, eabe7500. 10.1126/science.abe7500 34112668

[B87] Rojas A. P. Vo D. V. Mwangi L. Rehman S. Peiris A. N. (2020). Oncologic manifestations of Klinefelter syndrome. Hormones 19, 497–504. 10.1007/s42000-020-00241-7 33000452

[B88] Rougeulle C. Chaumeil J. Sarma K. Allis C. D. Reinberg D. Avner P. (2004). Differential histone H3 lys-9 and lys-27 methylation profiles on the X chromosome. Mol. Cell. Biol. 24, 5475–5484. 10.1128/MCB.24.12.5475-5484.2004 15169908 PMC419884

[B89] Rubin J. B. Abou-Antoun T. Ippolito J. E. Llaci L. Marquez C. T. Wong J. P. (2024). Epigenetic developmental mechanisms underlying sex differences in cancer. J. Clin. Invest. 134, e180071. 10.1172/JCI180071 38949020 PMC11213507

[B90] Sadagopan A. Nasim I. T. Li J. Achom M. Zhang C. Z. Viswanathan S. R. (2022). Somatic XIST activation and features of X chromosome inactivation in Male human cancers. Cell Syst. 13, 932–944.e5. 10.1016/j.cels.2022.10.002 36356577

[B91] Salama E. A. Adbeltawab R. E. El Tayebi H. M. (2020). XIST and TSIX: novel cancer immune biomarkers in PD-L1-overexpressing breast cancer patients. Front. Oncol. 9, 1459. 10.3389/fonc.2019.01459 31998636 PMC6966712

[B92] Santiwatana S. Mahachoklertwattana P. Limwongse C. Khlairit P. Pongratanakul S. Roothumnong E. (2018). Skewed X chromosome inactivation in girls and female adolescents with autoimmune thyroid disease. Clin. Endocrinol. 89, 863–869. 10.1111/cen.13857 30229980

[B93] Sathishkumar C. Prabu P. Mohan V. Balasubramanyam M. (2018). Linking a role of lncRNAs (long non-coding RNAs) with insulin resistance, accelerated senescence, and inflammation in patients with type 2 diabetes. Hum. Genomics 12, 41. 10.1186/s40246-018-0173-3 30139387 PMC6107963

[B94] Sauteraud R. Stahl J. M. James J. Englebright M. Chen F. Zhan X. (2021). Inferring genes that escape X-chromosome inactivation reveals important contribution of variable escape genes to sex-biased diseases. Genome Res. 31, 1629–1637. 10.1101/gr.275677.121 34426515 PMC8415373

[B95] Schoeftner S. Sengupta A. K. Kubicek S. Mechtler K. Spahn L. Koseki H. (2006). Recruitment of PRC1 function at the initiation of X inactivation independent of PRC2 and silencing. EMBO J. 25, 3110–3122. 10.1038/sj.emboj.7601187 16763550 PMC1500994

[B96] Scofield R. H. Wren J. D. Lewis V. M. (2025). The toll like receptor 7 pathway and the sex bias of systemic lupus erythematosus. Front. Immunol. 16, 1479814. 10.3389/fimmu.2025.1479814 40051623 PMC11882868

[B97] Shin H. Choi W. L. Lim J. Y. Huh J. H. (2022). Epigenome editing: targeted manipulation of epigenetic modifications in plants. Genes Genomics 44, 307–315. 10.1007/s13258-021-01199-5 35000141

[B98] Shoukat H. M. H. Ghous G. Tarar Z. I. Shoukat M. M. Ajmal N. (2020). Skewed inactivation of X chromosome: a cause of hemophilia manifestation in carrier females. Cureus 12, e11216. 10.7759/cureus.11216 33269146 PMC7704156

[B99] Sierra I. Pyfrom S. Weiner A. Zhao G. Driscoll A. Yu X. (2023). Unusual X chromosome inactivation maintenance in female alveolar type 2 cells is correlated with increased numbers of X-linked escape genes and sex-biased gene expression. Stem Cell Rep. 18, 489–502. 10.1016/j.stemcr.2022.12.005 PMC9968984 36638790

[B100] Silver D. P. Dimitrov S. D. Feunteun J. Gelman R. Drapkin R. Lu S. D. (2007). Further evidence for *BRCA1* communication with the inactive X chromosome. Cell 128, 991–1002. 10.1016/j.cell.2007.02.025 17350581

[B101] Soares M. Wemans I. S. Caldas P. da Rocha S. T. Grosso A. R. (2025). X-linked transcriptome dysregulation across immune cells in systemic lupus erythematosus. Biol. Sex. Differ. 16, 69. 10.1186/s13293-025-00750-3 40999523 PMC12466074

[B102] Souyris M. Cenac C. Azar P. Daviaud D. Canivet A. Grunenwald S. (2018). *TLR7* escapes X chromosome inactivation in immune cells. Sci. Immunol. 3, eaap8855. 10.1126/sciimmunol.aap8855 29374079

[B103] Spiering A. E. Groenheide P. J. Mokry M. Onland-Moret N. C. Civelek M. Reue K. (2026). Sex chromosomes and cardiovascular disease. Eur. J. Prev. Cardiol. 33, 316–326. 10.1093/eurjpc/zwaf224 40231569

[B104] Sun Y. Qian Y. Sun H. X. Chen M. Luo Y. Xu X. (2022). Case report: *de novo DDX3X* mutation caused intellectual disability in a female with skewed X-chromosome inactivation on the mutant allele. Front. Genet. 13, 999442. 10.3389/fgene.2022.999442 36299587 PMC9589230

[B105] Tao Z. Bu S. Liang L. Yang Y. She K. Lu F. (2023). Visual acuity-related outer retinal structural parameters on swept source optical coherence tomography and angiography in XLRS patients and carriers. Transl. Vis. Sci. Technol. 12, 7. 10.1167/tvst.12.12.7 PMC10702782 38054929

[B106] Tjalsma S. J. D. Hori M. Sato Y. Bousard A. Ohi A. Raposo A. C. (2021). H4K20me1 and H3K27me3 are concurrently loaded onto the inactive X chromosome but dispensable for inducing gene silencing. EMBO Rep. 22, e51989. 10.15252/embr.202051989 33605056 PMC7926250

[B107] Topa H. Benoit-Pilven C. Tukiainen T. Pietiläinen O. (2024). X-chromosome inactivation in human iPSCs provides insight into X-regulated gene expression in autosomes. Genome Biol. 25, 144. 10.1186/s13059-024-03286-8 38822397 PMC11143737

[B108] Wagenhäuser L. Rickert V. Sommer C. Wanner C. Nordbeck P. Rost S. (2022). X-chromosomal inactivation patterns in women with fabry disease. Mol. Genet. Genomic Med. 10, e2029. 10.1002/mgg3.2029 35971858 PMC9482401

[B109] Wang D. Tang L. Wu Y. Fan C. Zhang S. Xiang B. (2020). Abnormal X chromosome inactivation and tumor development. Cell. Mol. Life Sci. 77, 2949–2958. 10.1007/s00018-020-03469-z 32040694 PMC11104905

[B110] Weber C. M. Harris M. N. Zic S. M. Sangha G. S. Arnold N. S. Dluzen D. F. (2023). Angiotensin II increases oxidative stress and inflammation in female, but not Male, endothelial cells. Cell. Mol. Bioeng. 16, 127–141. 10.1007/s12195-023-00762-2 37096068 PMC10121986

[B111] Werner J. M. Ballouz S. Hover J. Gillis J. (2022). Variability of cross-tissue X-chromosome inactivation characterizes timing of human embryonic lineage specification events. Dev. Cell 57, 1995–2008.e5. 10.1016/j.devcel.2022.07.007 35914524 PMC9398941

[B112] Wufuer A. Mijiti P. Abudusalamu R. Dengfeng H. Jian C. Jianhua M. (2019). Blood pressure and collateral circulation in acute ischemic stroke. Herz 44, 455–459. 10.1007/s00059-018-4691-5 29556676

[B113] Xiong Y. Zhang Y. Xiong S. Williams-Villalobo A. E. (2020). A glance of p53 functions in brain development, neural stem cells, and brain cancer. Biol. (Basel) 9, 285. 10.3390/biology9090285 32932978 PMC7564678

[B114] Yin S. Zhong N. Agrawal S. Feng B. Pradhan S. Jobalia N. K. (2025). Single- cell genomic copy number evolution reveals frequent loss of the Y chromosome in esophageal adenocarcinoma. bioRxiv [preprint]. 10.1101/2025.09.14.676175v1

[B115] Youness A. Cenac C. Faz-López B. Grunenwald S. Barrat F. J. Chaumeil J. (2023). *TLR8* escapes X chromosome inactivation in human monocytes and CD4^+^ T cells. Biol. Sex. Differ. 14, 60. 10.1186/s13293-023-00544-5 37723501 PMC10506212

[B116] Zhang Y. Li X. Gibson A. Edberg J. Kimberly R. P. Absher D. M. (2020). Skewed allelic expression on X chromosome associated with aberrant expression of XIST on systemic lupus erythematosus lymphocytes. Hum. Mol. Genet. 29, 2523–2534. 10.1093/hmg/ddaa131 32628254

[B117] Zhang P. Munier J. J. Wiese C. B. Vergnes L. Link J. C. Abbasi F. (2024). X chromosome dosage drives statin-induced dysglycemia and mitochondrial dysfunction. Nat. Commun. 15, 5571. 10.1038/s41467-024-49764-2 38956041 PMC11219728

[B118] Zhang C. M. Ge Z. B. Zhou H. H. Wei M. X. Ding X. Y. Lin Z. Z. (2025). Sex chromosomes/hormones and the tumor microenvironment of non-reproductive cancers. Front. Immunol. 16, 1642956. 10.3389/fimmu.2025.1642956 41019050 PMC12463827

[B119] Zito A. Roberts A. L. Visconti A. Rossi N. Andres-Ejarque R. Nardone S. (2023). Escape from X-inactivation in twins exhibits intra- and inter-individual variability across tissues and is heritable. PLoS Genet. 19, e1010556. 10.1371/journal.pgen.1010556 36802379 PMC9942974

[B120] Zitzmann M. Sansone A. Jannini E. A. Jones H. Ferlin A. Lunenfeld B. (2025). Recommendations for diagnosis and treatment of the aging Male with Klinefelter syndrome. Aging Male 28, 2519035. 10.1080/13685538.2025.2519035 40536882

[B121] Żylicz J. J. Bousard A. Žumer K. Dossin F. Mohammad E. da Rocha S. T. (2019). The implication of early chromatin changes in X chromosome inactivation. Cell 176, 182–197.e23. 10.1016/j.cell.2018.11.041 30595450 PMC6333919

