# Correction: The impact of X chromosome inactivation on human health

**DOI:** 10.3389/fgene.2026.1898696

**Published:** 2026-06-29

**Authors:** Junyi Gu, Qian Chen

**Affiliations:** 1 Department of Reproductive Medicine, The First Affiliated Hospital of Xi’an Jiaotong University, Xi’an, Shaanxi, China; 2 School of Medicine, Xi’an Jiaotong University, Xi’an, Shaanxi, China

**Keywords:** health impact, sex differences, therapeutic strategies, X chromosome inactivation, XCI escape, XCI skewing

The reference for “Barakat et al., 2011” was erroneously written as “Barakat, T. S., Gunhanlar, N., Pardo, C. G., Achame, E. M., Ghazvini, M., Boers, R., et al. (2011). RNF12 activates xist and is essential fo.R. X Chromosome Inactivation. *PLoS Genet*. 7, e1002001. doi:10.1371/journal.pgen.1002001”. It should be “Barakat, T. S., Gunhanlar, N., Pardo, C. G., Achame, E. M., Ghazvini, M., Boers, R., et al. (2011). RNF12 activates Xist and is essential for X chromosome inactivation. *PLoS Genet*. 7, e1002001. doi:10.1371/journal.pgen.1002001”.

The reference for “Carrette et al., 2018” was erroneously written as “Carrette, L. L. G., Wang, C.-Y., Wei, C., Press, W., Ma, W., Kelleher, R. J., et al. (2018). A mixed modality approach towards Xi reactivation for Rett syndrome and other X-linked disorders. *Proc. Natl. Acad. Sci. USA*. 115, E668–E675. doi:10.1073/pnas.1715124115”. It should be “Carrette, L. L. G., Wang, C.Y., Wei, C., Press, W., Ma, W., Kelleher, R. J., et al. (2018). A mixed modality approach towards Xi reactivation for Rett syndrome and other X-linked disorders. Proc. Natl. Acad. Sci. USA. 115, E668–E675. doi:10.1073/pnas.1715124115”.

The reference for “Leonardi et al., 2023” was erroneously written as “Leonardi, E., Aspromonte, M.C., Drongitis, D., Bettella, E., Verrillo, L., Polli, R., et al. (2022). Expanding the genetics and phenotypic spectrum of Lysine-specific demethylase 5C (KDM5C): a report of 13 novel variants. *Eur J Hum Genet*. 31: 202-215. doi: 10.1038/s41431-022-01233-4”. It should be “Leonardi, E., Aspromonte, M.C., Drongitis, D., Bettella, E., Verrillo, L., Polli, R., et al. (2023). Expanding the genetics and phenotypic spectrum of Lysine-specific demethylase 5C (KDM5C): a report of 13 novel variants. *Eur J Hum Genet*. 31: 202-215. doi: 10.1038/s41431-022-01233-4”.

The reference for “McHugh et al., 2015” was erroneously written as “McHugh, C. A., Chen, C. K., Chow, A., Surka, C. F., Tran, C., McDonel, P., et al. (2025). The xist lncRNA interacts directly with SHARP to silence transcription through HDAC3. *Nature* 521, 232–236. doi:10.1038/nature14443”. It should be “McHugh, C. A., Chen, C. K., Chow, A., Surka, C. F., Tran, C., McDonel, P., et al. (2015). The xist lncRNA interacts directly with SHARP to silence transcription through HDAC3. *Nature* 521, 232–236. doi:10.1038/nature14443”.

The reference for “Salama et al., 2020” was erroneously written as “Salama, E. A., Adbeltawab, R. E., and El Tayebi, H. M. (2019). XIST and TSIX: novel cancer immune biomarkers in PD-L1-overexpressing breast cancer patients. *Front. Oncol*. 9, 1459. doi:10.3389/fonc.2019.01459”. It should be “Salama, E. A., Adbeltawab, R. E., and El Tayebi, H. M. (2020). XIST and TSIX: novel cancer immune biomarkers in PD-L1-overexpressing breast cancer patients. *Front. Oncol*. 9, 1459. doi:10.3389/fonc.2019.01459”.

The reference for “Werner et al., 2022” was erroneously written as “Werner, J. M., Ballouz, S., Hover, J., and Gillis, J. (2023). Variability of cross-tissue X-chromosome inactivation characterizes timing of human embryonic lineage specification events. *Dev. Cell* 57, 1995–2008.e5. doi:10.1016/j.devcel.2022.07.007”. It should be “Werner, J. M., Ballouz, S., Hover, J., and Gillis, J. (2022). Variability of cross-tissue X-chromosome inactivation characterizes timing of human embryonic lineage specification events. *Dev. Cell* 57, 1995–2008.e5. doi:10.1016/j.devcel.2022.07.007”.

A correction has been made to Section 4.1, first paragraph: Moreover, in TNBC and HCC, escape gene-associated signatures have been employed to construct prognostic models for predicting hypoxia, immune evasion, and treatment outcomes (Ba et al., 2025; Yin et al., 2025). (Ba et al., 2025; Yin et al., 2025). The correct content should be: “Moreover, in TNBC and HCC, escape gene-associated signatures have been employed to construct prognostic models for predicting hypoxia, immune evasion, and treatment outcomes (Ba et al., 2025; Yin et al., 2025)”.

A correction has been made to Section 2.1, second paragraph: RNA plays a pivotal role in this process by interacting with the SPlit ENds (SPEN) protein, receptor corepressor (NCoR), silencing mediator of retinoic acid and thyroid hormone receptor (SMRT), and nucleosome remodeling deacetylase (NuRD) to mediate histone deacetylation (Dossin et al., 2020; Jachowicz et al., 2022; McHugh et al., 2025; Żylicz et al., 2019). The correct content should be: “RNA plays a pivotal role in this process by interacting with the SPlit ENds (SPEN) protein, receptor corepressor (NCoR), silencing mediator of retinoic acid and thyroid hormone receptor (SMRT), and nucleosome remodeling deacetylase (NuRD) to mediate histone deacetylation (Dossin et al., 2020; Jachowicz et al., 2022; McHugh et al., 2015; Żylicz et al., 2019)”.

A correction has been made to Section 3.2, third paragraph: As a histone demethylase, loss of *KDM5C* function not only affects chromatin structure but may also interact with XCI skewing by regulating the expression of other X-linked gene (Leonardi et al., 2022). The correct content should be: “As a histone demethylase, loss of *KDM5C* function not only affects chromatin structure but may also interact with XCI skewing by regulating the expression of other X-linked gene (Leonardi et al., 2023)”.

A correction has been made to Section 3.4, second paragraph: The epigenetic landscape shaped by XCI skewing also modulates immune responses in the tumor microenvironment, contributing to sex-specific differences in cancer immunity and therapeutic responses (Rojas et al., 2020; Salama et al., 2019). The correct content should be: “The epigenetic landscape shaped by XCI skewing also modulates immune responses in the tumor microenvironment, contributing to sex-specific differences in cancer immunity and therapeutic responses (Rojas et al., 2020; Salama et al., 2020)”.

A correction has been made to Section 4.1, third paragraph: Another study found that XCI ratios are highly consistent between the liver and whole blood, but exhibit greater variability in brain tissue, reflecting differences in cell numbers during lineage specification in embryonic development (Werner et al., 2023). The correct content should be: “Another study found that XCI ratios are highly consistent between the liver and whole blood, but exhibit greater variability in brain tissue, reflecting differences in cell numbers during lineage specification in embryonic development (Werner et al., 2022)”.

A correction has been made to Introduction, third paragraph: In the realm of oncology, irregular XCI patterns and evasion from inactivation have been associated with tumor development and differences in cancer susceptibility based on sex (McHugh et al., 2025). The correct content should be: “In the realm of oncology, irregular XCI patterns and evasion from inactivation have been associated with tumor development and differences in cancer susceptibility based on sex (Sadagopan et al., 2022)”.

The reference for “Tjalsma et al., 2021” was erroneously written as “Tjalsma, S.J.D., Hori, M., Sato, Y., Bousard, A., Ohi, A., Raposo, A.C., et al. (2021). H4K20me1 and H3K27me3 are concurrently loaded onto the inactive X chromosome but dispensable for inducing gene silencing. EMBO Rep. 22: e5198. doi:10.15252/embr.202051989”. It should be “Tjalsma, S.J.D., Hori, M., Sato, Y., Bousard, A., Ohi, A., Raposo, A.C., et al. (2021). H4K20me1 and H3K27me3 are concurrently loaded onto the inactive X chromosome but dispensable for inducing gene silencing. EMBO Rep. 22: e51989. doi:10.15252/embr.202051989”.

The original article has been updated.

